# MRI-Based Image Signal-to-Noise Ratio Enhancement with Different Receiving Gains in K-Space

**DOI:** 10.3390/s21165296

**Published:** 2021-08-05

**Authors:** Lin Wu, Shuang Zhang, Tao Zhang

**Affiliations:** 1 School of Life Science and Technology, University of Electronic Science and Technology of China, Chengdu 611731, China; neuwulin@163.com (L.W.); zhangshuanghua1@126.com (S.Z.); 2 College of Computer Science, Neijiang Normal University, Neijiang 641112, China; 3 High Field Magnetic Resonance Brain Imaging Laboratory of Sichuan, University of Electronic Science and Technology of China, Chengdu 611731, China; 4 Shanghai Electric Group Company Limited, Shanghai 200240, China

**Keywords:** MRI, receiving gain, signal-to-noise ratio, k-space, phase encoding

## Abstract

Echo signals in different regions in the k-space of magnetic resonance imaging (MRI) data possess different amplitudes. The signal-to-noise ratio (SNR) of a received signal can be improved by differentially setting the receiving gain (RG) parameter in different areas of the k-space. Previously, the k-space data splicing method and the gain normalization implementation method were not specifically investigated; however, this study focuses on this aspect. Specifically, to improve the SNR, three RGs and MRI scans are herein designed for each gain parameter using the gradient echo sequence to obtain one group of k-space data. Subsequently, the three groups of experimental k-space data obtained using MRI scans are spliced into one group of k-space data. For the splicing process, a method for gain and phase correction and compensation is developed that normalizes different RG parameters in the k-space. The experimental results indicate that the developed methods improve the SNR by 5–13%. When the RGs are set to other combinations, the k-space data splicing and gain normalization methods presented in this paper are still applicable.

## 1. Introduction

Signal acquisition research has thus far focused on the maximum signal-to-noise ratio (SNR) at individual amplitude points to prevent saturation under a fixed receiving gain (RG). Several researchers have attempted to improve the SNR by reducing the receiver noise [1,2,3,4,5,6,7,8]. When compared with analog receivers, digital receivers can reduce the noise of nuclear magnetic resonance (NMR) and magnetic resonance imaging (MRI), thereby improving the SNR [1,2,3,4]. For conventional MRI digital receivers (3T), analog-to-digital convertors (ADCs) with a conversion rate of 100 MHz are typically used for under-sampling and analog-to-digital signal conversion [5]. Under the normal temperature operating mode, the SNR of a system does not improve even if the ADC sampling rate exceeds 100 MHz, owing to the impact of physiological noise from the human body [6]. Masoud et al. proposed a 20 GHz superconducting ADC to sample the MRI signals in an ultra-low-temperature environment and reduce the impact of human physiological noise, thereby improving the SNR [6]. However, their proposed superconducting ADC requires ultra-low temperatures to function, which limits its practical application in MRI. Diu and A.T. used redundant information detection to achieve a lower sampling noise and a higher sampling resolution for the internal design of an ADC, further improving the quantization SNR [7,8]. However, because this method involves chip design, its implementation is more complex. In addition to the sampling rate of the ADC being increased, the SNR of the sampling signal can also be improved by increasing the number of bits of the ADC. However, this requires redesigning the spectrometer hardware itself. Therefore, this approach will yield expensive and complex commercial MR systems [9,10].

Because the amplitude of the free induction decay (FID) signal of NMR fluctuates greatly in terms of duration, the method for reducing the receiver noise under a fixed RG proposed in the literature [1,2,3,4,5,6,7,8] leverages the entire resolution of an ADC for the part with a larger amplitude in the signal; however, it does not consider the dynamic range of the signal across the entire period during the quantization process. Although measures have been taken to reduce the sampling noise, the parts of the signal with smaller amplitudes still cannot leverage the full resolution of an ADC [1,2,3,4,5,6,7,8,11], meaning that the maximum possible SNR cannot be achieved. In 2011, Takeda et al. proposed a solution, namely, Apodization after Receiver Gain Increment during Ongoing Sequence with Time (APRICOT). By means of dynamically adjusting the RG, the solution not only increases the SNR of the part with a larger amplitude in the FID signal, but also the receiver gain of the part with a smaller amplitude. Therefore, the small peak signal masked by digital noise can be identified [12]. However, for best results, a series of parameters, including the preamplifier noise index, signal accumulation number, and signal spectrum width, must be matched simultaneously because APRICOT employs an open-loop RG control pattern. Therefore, it is difficult to accurately control the RG via this method. Inspired by the propositions of Takeda et al., Jouda et al. proposed an improved closed-loop RG adjustment method. By monitoring the signal amplitude in real time and adjusting the RG during signal acquisition, they accurately recovered the quantized signals of an ADC. Their method improved the SNR of the FID over the entire period via dynamic adjustment of the RG. However, it requires an accurate closed-loop RG control circuit, which complicates the circuit design [11].

Each NMR scan can only obtain one line of FID data; conversely, with MRI scanning, a group of k-space data can be obtained, in which the k-space contains multiple phase-encoded line data. In turn, each phase-encoded line represents an echo signal. In the echo signal of the same phase-encoded line in the k-space, the amplitude of a low-frequency signal is larger than that of a high-frequency signal. Setting a fixed RG according to the point with the largest amplitude in the echo will not allow the dynamic range of the whole period echo signal to reach the optimum [11,13,14,15]. Kose et al. [13] used the nonlinear RG method utilized in a simulation to compress the low-frequency component of an echo signal and expand the high-frequency component, which greatly improved the SNR. Based on this simulation, Siemens proposed a nonlinear RG scheme [14] and used it to design a type of signal amplitude compression expander, which was installed in the front-end of the ADC device in an MRI receiver. However, this scheme needs to not only meet the dynamic range and compression requirements, but also consider the nonlinear compression signal recoverability, voltage offset, temperature stability, and other factors. Therefore, its implementation poses great challenges to engineers.

Otake et al. [15] proposed a method to improve the SNR of MRI through parallel acquisition. In their scheme, two acquisition circuits were designed to detect the coil induction signal in parallel, one of which was a high-RG acquisition circuit, whereas the other was a low one. The former could ensure that the part of the echo signal with a smaller amplitude reached a larger dynamic range, while the latter could prevent saturation of the part of the echo signal with a larger amplitude. Through analysis, they demonstrated the easy implementation of the method and its ability to improve the SNR of an MRI signal by approximately 6 dB. However, the method needed two acquisition circuits to acquire the coil induction signal of each channel in parallel, which increased the material cost and system complexity of the MRI receiver. Otake et al. [15] also proposed using a histogram to count the high and low gain and phase differences but did not specify the data sample selection method. In recent years, significant progress has been made in improving image SNR through MR image post-processing algorithms [16,17,18]. If the research focus is shifted from image reconstruction to signal-receiving hardware, optimizing the control parameters of the receiving hardware would also be a good option to improve the SNR of the sampled signal.

References [13,14,15] only considered methods to improve the dynamic range of a single echo signal in the k-space. However, among all the phase-encoding lines in the MRI k-space, each line has a distinct echo-signal amplitude. Larger phase-encoding lines have smaller signal amplitudes than those of smaller phase-encoding lines. Therefore, in the process of gain setting, the dynamic range of the entire echo signal cannot be optimized by setting a fixed RG based on the premise that the maximum echo amplitude of all phase-encoding lines in the whole k-space does not saturate [11]. Elliot et al. and Oh et al. found that optimizing the RG parameters of different regions in the MRI k-space without changing the MRI receiver circuit could also increase the dynamic range of the signal [19,20]. Elliot et al. set six RG parameters, with each one set in increments of 6 dB. The sequence was scanned once under every RG parameter, and a group of k-space data was obtained every time. Thus, six groups of k-space data were spliced into one group of k-space data, and a three-dimensional image was obtained using the Fourier transform. When compared with that of the image reconstructed from k-space data at the lowest gain, the quality of the image reconstructed from spliced k-space data was found to have improved [19]. However, that report only mentioned the need for a correction system that could rectify the differences among different gain parameters and did not elaborate on the correction method.

Oh et al. improved the SNR of an MRI image by optimizing the RG parameters of each phase-encoding line in a k-space [20]. In their method, the maximum M(*n*) of the echo signal of each phase-encoding line was searched, and the optimal gain $g\left(n\right)=\frac{Sm}{M\left(n\right)}$ of each line was determined, where S*m* is the maximum of the entire k-space signal, and *n* is the line number of the phase-encoding line. Their simulation results showed that the optimal sampling bit-width of an ADC would require at least 19 bits under a fixed receiver gain, but only greater than 15 bits under a variable gain. Thus, they significantly reduced the demand for ADC sampling bit bandwidth. As the MRI platform used by Oh et al. did not support the dynamic change in the RG parameters during sequence operation, they used the ideas of Elliot et al. to design the actual experimental paradigm. They obtained a group of k-space data under each gain parameter setting, and then spliced the k-space data obtained under different RG parameters. They stated that different gain parameters must be normalized to the same RG parameter but did not refer to any specific implementation method.

Given this context, this paper proposes a gain-difference correction method that is applicable when different regions of the k-space are assigned different RG parameters. Simultaneously, based on the experimental data, we detail a method to correct and compensate for the RG parameter in each area of the k-space.

The remainder of this paper is organized as follows. Section 2 presents the experimental scheme, and Section 3 details the verification of the method through an experiment. Finally, Section 4 presents the analysis of the experimental results and their interpretation.

## 2. Experimental Scheme

To improve the SNR of the received signal, we designed three RGs in this study, and a gradient echo (GRE) sequence scan on each RG parameter was conducted to obtain three groups of k-space data. Then, these three groups were spliced into one group. In the splicing process, data obtained under different k-space gain parameters are normalized by the amplitude under the maximum RG parameter. Herein, we also present the methods to correct and compensate for the gain and phase in the normalization process. An SNR comparison between the images reconstructed from the spliced k-space data and the k-space data obtained under the lowest RG was further performed to verify the effectiveness of the proposed k-space data splicing and gain normalization methods.

A commercial 1.5T MR scanner with 16 parallel receiving channels (Alltech Medical Systems LLC, Chengdu, China) was used to comprehensively verify the effectiveness of the research method proposed in this paper. An 8-channel receiver coil was used to conduct the MRI imaging experiment. Additionally, the effectiveness of the proposed method was also verified based on the imaging SNR enhancement effect of each channel. The receiver coil in the system adopts an 8-channel phased array head coil (crown structure design; the upper and lower parts are inseparable), with the eight units of the head coil being similar in size and shape and evenly distributed. This design helps to improve the SNR and uniformity. The coil dimensions were as follows: length = 340 mm and diameter = 240 mm. To calculate the SNR of MRI images more conveniently, a homogeneous water model with a simple internal structure was used as the imaging sample in this study. Homogeneous phantoms (spherical, 200 mm diameter; composition: water (92%), NaCl (5%), and NiSO_4_·6(H_2_O) (3%)) were used as imaging samples.

Algorithm 1 shows the main steps of the experiment designed in this paper. The commercial 1.5T MR scanner software interface used in this study has a menu that allows the user to set the RG. The RG parameter is used to control the gain of the MRI spectrometer receiver. The larger this parameter value is, the greater the magnification of the received signal by the spectrometer receiver will be. The parameters for sequence scanning measurement were set as follows: RG: τ, τ − 6, and τ − 12 dB. The attenuation constants τ = 6 dB and 12 dB were obtained via a programmable digital-step attenuation controller in the receiver. TR: 68 ms, TE: 5.2 ms, NEX = 1, receiving channel numbers = 8, excitation slices = 4, scan matrix: 220 × 440, image view matrix: 220 mm × 220 mm, Flip angle: 70°, dw: 8 µs, thickness: 5.0 mm, slice gap: 1.0 mm. During data acquisition, each time an RG control parameter was adjusted, a sequence scan was performed to obtain a group of the original k-space data (8 × 4 × 220 × 440, floating point data).
**Algorithm 1:** System execution procedure.**Result:** Spliced k-space data with enhanced SNR **Input:** **Init:** Start GRE sequence scanning and configure the RG parameters as τ, τ − 6, and τ − 12 dB; **Step 1:** Obtain 3 sets of k-space data: K_τ_ obtained under RG = τ, K_τ−6_ obtained under RG = τ − 6, K_τ−12_ obtained under RG = τ − 12; **Step 2:** Correct the gain difference and phase difference for K_τ_ and K_τ−6_, Correct the gain difference and phase difference for K_τ−6_ and K_τ−12_, Calculate the gain difference and phase difference for K_τ_ and K_τ−12_; **Step 3:** Extract K_τ_^1^, K_τ−6_^2^, and K_τ−12_^3^ from K_τ_, K_τ−6_, and K_τ−12_, respectively to obtain a set of spliced k-space data. **Step 4:** Compensate for the gain difference between K_τ_^1^, K_τ−6_^2^, and K_τ−12_^3^; **Step 5:** Obtain image data by Fourier transform of the spliced k-space data. **Output:** SNR of the image obtained by Fourier transform of the spliced k-space data

**Figure 1:**
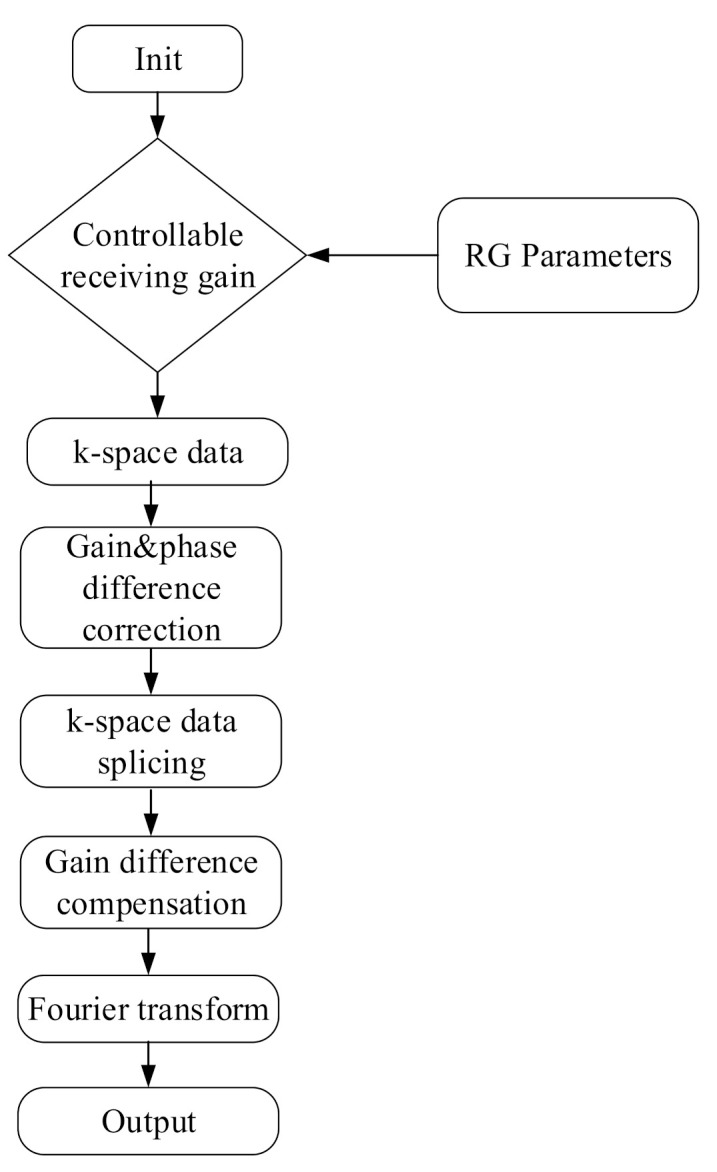
System execution flowchart.

The k-space data (1 × 1 × 220 × 440) with the first channel and the first slice were extracted to obtain three groups of memory-filling matrix data, K_τ_, K_τ–6_, and K_τ–12_, with RGs of τ, τ − 6, and τ − 12 dB, respectively. The RG corresponds to the amplification factor of the received k-space echo signal in the MR system. Theoretically, the RG differences corresponding to K_τ_ and K_τ−6_ are τ dB − (τ − 6 dB) = 6 dB, i.e., the difference between the amplification factors was 10^(6/20)^ ≈ 2 times, and the difference between the RGs corresponding to K_τ_ and K_τ–12_ was τ dB − (τ − 12 dB) = 12 dB, i.e., the difference between the amplification factors is 10^(12/20)^ ≈ 4 times. A data space of a spliced image was constructed by analyzing the gain and phase differences of the three datasets. Thus, an image could be obtained through the Fourier transform of the spliced data space. Figure 1 illustrates the execution process of the system.

## 3. Experimental Verification

The phase-encoding line number filling the memory space of the k-space was denoted by m. To describe the three RG expressions simultaneously in the same image, K_τ_ was first divided into three areas, namely K_τ_^1^, K_τ_^2^, and K_τ_^3^, respectively, where K_τ_^1^ is *m* = 1–36, *m* = 185–220; K_τ_^2^ is *m* = 37–72, *m* = 149–184; and K_τ_^3^ is *m* = 73–148. Similarly, K_τ−6_ and K_τ−12_ were also divided into three areas. To normalize the data of the three areas to the same RG level, their gain and phase differences must be corrected.

### 3.1. Data Splicing

Under the same RG in all the areas in the k-space, the amplitude of the echo signal meets the following criteria.

K_()_^1^ < K_()_^2^ < K_()_^3^ (1)

**Figure 2:**
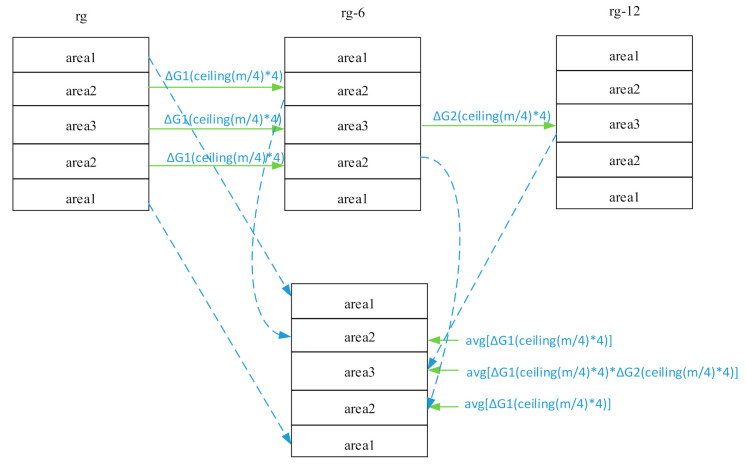
k-space data splicing and gain correction.

Assuming K is the spliced data space, we considered K_τ_^1^ of K_τ_ as K^1^ of K, K_τ−6_^2^ of K_τ−6_ as K^2^ of K, K_τ−12_^3^ of K_τ−12_ as K^3^ of K. Among them, the echo signal of K^1^ was obtained under the maximum RG to ensure that the echo signal of the K^1^ area reached the maximum signal dynamic range; the echo signal of K^3^ was obtained under the minimum RG to ensure that the echo signal of the K^3^ area did not overflow (shown in Figure 2).

### 3.2. Gain Difference Correction

In K_τ_^2^ and K_τ_^3^ of K_τ_, and K_τ−6_^2^ and K_τ−6_^3^ of K_τ−6_, the same four lines of data were extracted in order every time. The middle *N* data with the phase-encoding line number ceil(m/4) × 4 were defined as $\delta_{\tau i},$ $\delta_{\left(\tau -6\right)i}$, i=1,2,⋯,N; where $\delta_{\tau i}$ and $\delta_{\left(\tau -6\right)i}$ are absolute values. The extraction order is as follows:

(2) $$\alpha_{i}=\frac{\delta_{\tau i}}{\delta_{\left(\tau -6\right)i}},\;i=1,2,\cdots ,N;$$

(3) βτ,τ−6=∑i=1NαiN

$\beta_{\tau ,\tau -6}$, the gain difference factor of the corresponding phase-encoding lines of K_τ_ and K_τ−6_, was $\Delta G1\left(\mathrm{ceil}\left(m/4\right)\times 4\right)$. In K_τ_^2^ and K_τ_^3^ of K_τ_, and K_τ−6_^2^ and K_τ−6_^3^ of K_τ−6_, the sum Δ*G*1(ceil(m/4) × 4) of all lines and their average value were used to obtain avg[Δ*G*1(ceil(m/4) × 4)]. The gain difference factor in K_τ_^2^ and K_τ_^3^ of K_τ_ and K_τ−6_^2^ and K_τ−6_^3^ of K_τ−6_ was avg[Δ*G*1(ceil(m/4) × 4)].

In K_τ−6_^3^ of K_τ−6_ and K_τ−12_^3^ of K_τ−12_, the same four lines of data were extracted in order each time, and the middle N data with the phase-encoding line number ceil(m/4) × 4 were defined as $\delta_{\left(\tau -6\right)i}$, $\delta_{\left(\tau -12\right)i}$, i=1, 2,⋯,N; where $\delta_{\left(\tau -6\right)i}$ and $\delta_{\left(\tau -12\right)i}$ are absolute values. The extraction order is as follows:

(4) $$\alpha_{i}=\frac{\delta_{\left(\tau -6\right)i}}{\delta_{\left(\tau -12\right)i}},\;i=1,2,\cdots ,N;$$

(5) βτ−6,τ−12=∑i=1NαiN

$\beta_{\tau -6,\tau -12}$, the gain difference factor of the corresponding phase-encoding lines of K_τ−6_^3^ and K_τ−12_^3^, was $\Delta G2\left(\mathrm{ceil}\left(m/4\right)\times 4\right)$. Assuming $\beta_{\tau ,\tau -12}$ to be the gain difference factor of the corresponding phase-encoding lines in K_τ_^3^ and K_τ−12_^3^, we derive

(6) $$\beta_{\tau ,\tau -12}=\;\Delta G1\left(\mathrm{ceil}\left(m/4\right)\times 4\right)\times \Delta G2\left(\mathrm{ceil}\left(m/4\right)\times 4\right).$$

In K_τ_^3^ and K_τ−12_^3^, $\beta_{\tau ,\tau -12}$ of all lines were summed and then averaged to obtain $\mathrm{avg}[\Delta G1\left(\mathrm{ceil}\left(m/4\right)\times 4\right)\times \Delta G2\left(\mathrm{ceil}\left(m/4\right)\times 4\right)$, which was the final gain difference factor in K_τ_^3^ and K_τ−12_^3^.

According to this method, the gain difference factors of K_τ_ (K_τ_^2^ and K_τ_^3^) and K_τ−6_ (K_τ−6_^2^ and K_τ−6_^3^) and K_τ_^3^ and K_τ−12_^3^ were 2.1173 and 3.9736, respectively.

**Figure 3:**
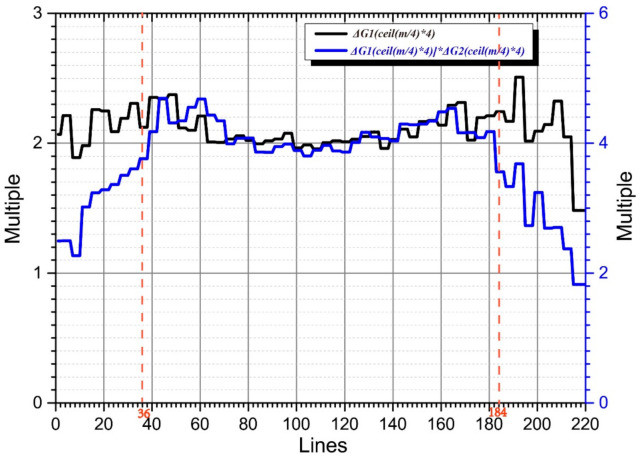
Gain difference factor calculation.

In Figure 3, the black curve represents the amplitude ratios of K_τ_ and K_τ−6_, and the blue curve represents those of K_τ_ and K_τ−12_. The signal amplitudes of lines 37 to 184 of K_τ_, K_τ−6_, and K_τ−12_ are all distributed above the noise plane. Therefore, in this region, the amplitude ratios are all approximately equal to their theoretical values (2 and 4, respectively).

Most of the effective components of the echo signals in lines 1–36 and 185–220 of K_τ−6_ and K_τ−12_ were distributed below the noise plane. Because the amplitude was mainly determined by the noise amplitude, the amplitude ratios of K_τ−6_ and K_τ−12_ in this area tends to one. As the line number moves to the middle region, the echo-signal amplitude gradually rises above the noise plane, and its amplitude ratio approaches two. The echo amplitudes of lines 1–36 and 185–220 of K_τ_ and K_τ−6_ were above the noise plane, and their amplitude ratios were approximately two. The blue curve values corresponding to lines 1–36 and 185–220 were composed of the amplitude ratios of K_τ−6_ and K_τ−12_, multiplied by those of K_τ_ and K_τ−6_. As the line number migrated to the middle region, the blue curve’s value gradually approached four.

### 3.3. Gain Compensation

Assuming that the spliced data space is K, we took K_τ_^1^ of K_τ_ as K^1^ of K, and K_τ−6_^2^ of K_τ−6_ as K^2^ of K. We obtained K^2*^ by multiplying the data amplitude of each line of K^2^ by $\mathrm{avg}[\Delta G1\left(\mathrm{ceil}\left(m/4\right)\times 4\right)$]. We took K_τ−12_^3^ of K_τ−12_ as K^3^ of K and obtained K^3*^ by multiplying the data amplitude of each line of K^3^ by $\mathrm{avg}[\Delta G1\left(\mathrm{ceil}\left(m/4\right)\times 4\right)\times \Delta G2\left(\mathrm{ceil}\left(m/4\right)\times 4\right)$]. Then, the k-space comprising K^1^, K^2*^, and K^3*^ is the compensated k-space.

### 3.4. Phase Correction

**Figure 4:**
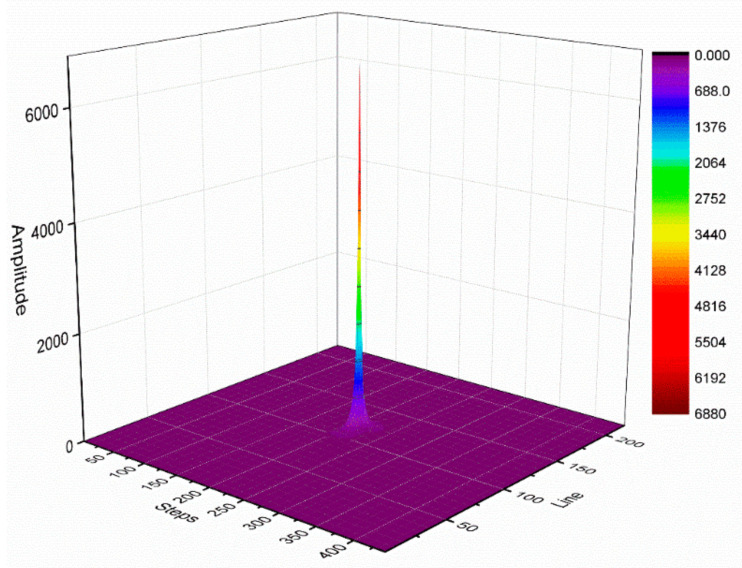
k-space signal amplitude distribution diagram.

Figure 4 illustrates the k-space signal amplitude distribution with a total of 220 phase-encoding lines and 440 frequency-encoding steps. The k-space central area data have a higher amplitude value, which describes the general contour information of the corresponding homogeneous water model image. The surrounding area data have a lower amplitude value, which describes the detailed information of the corresponding homogeneous water model image. On the one hand, the central area has a higher signal amplitude value than the surrounding region and is less affected by noise. Therefore, the phase difference of the corresponding position signal in the central area tends to be stable. On the other hand, the area surrounding the k-space has a low signal amplitude value and is more affected by noise. This would cause the phase difference of the signals from the surrounding area to considerably fluctuate. When analyzing the phase difference, we considered the middle *N* steps of each phase-encoding line for the analysis. In this study, we assumed *N* = 120.

**Figure 5:**
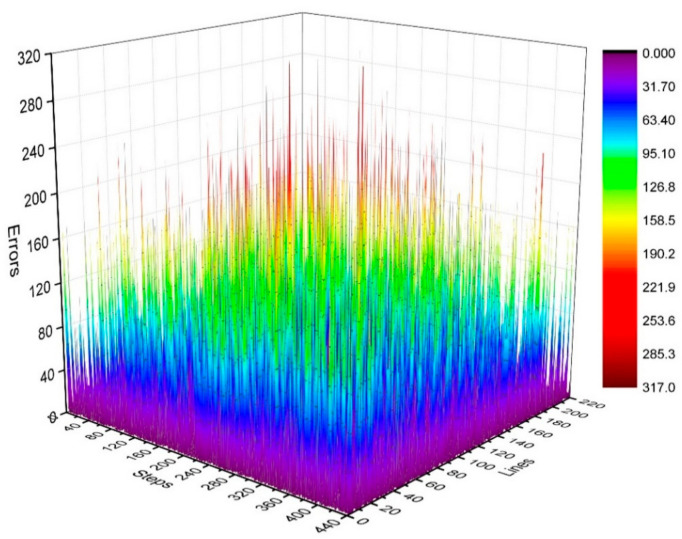
Three-dimensional statistical diagram of phase difference (error) distributions of K_τ_ and K_τ−6_.

**Figure 6:**
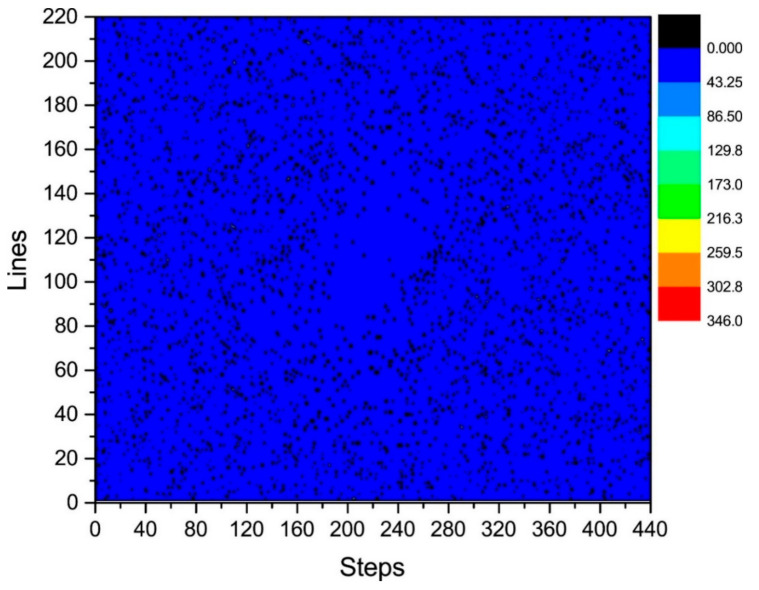
Statistical diagram of the phase difference (error) of K_τ_ and K_τ−6_.

In K_τ_^2^ and K_τ_^3^ of K_τ_, and K_τ−6_^2^ and K_τ−6_^3^ of K_τ−6_, we defined the phases of the *N* steps in the middle of each phase-encoding line as $\varepsilon_{\tau ij}$ and $\varepsilon_{\left(\tau -6\right)ij}$, where i is the number of phase-encoding lines, i=37, 38,…, 184, j=1, 2,⋯,N. The order is as follows:

(7) $$\gamma_{ij}=\varepsilon_{\tau ij}-\varepsilon_{\left(\tau -6\right)ij}$$

where i=37,  38,…, 184 and j=1,2,⋯,N. We calculated the distribution of $\gamma_{ij}$ in the k-space, as shown in Figure 5 and Figure 6.

Figure 5 illustrates the phase difference (error) distributions of the positions of K_τ_ and K_τ−6_ in degrees (°).

Figure 6 illustrates a plane diagram of the statistics of the phase difference (error) between signals at the same positions of K_τ_ and K_τ−6_.

By analyzing the regularities in the phase difference distribution in Figure 4, Figure 5 and Figure 6, we found that the signal amplitude value in the surrounding area of the k-space is low, and the signal is greatly influenced by noise. Therefore, the phase difference of the signal in the area surrounding the k-space fluctuates greatly. The central area has a higher signal amplitude, and the signal is less affected by noise. Therefore, the phase difference of the corresponding position signal in the central area tends to be stable (the phase difference approaches zero). As the signal from the central area is less affected by noise, the phase difference of the corresponding position signal in the area represents the phase difference of the corresponding position signal in the entire k-space. Therefore, the phase differences of all corresponding position signals of K_τ_ and K_τ−6_ were considered to be zero.

**Figure 7:**
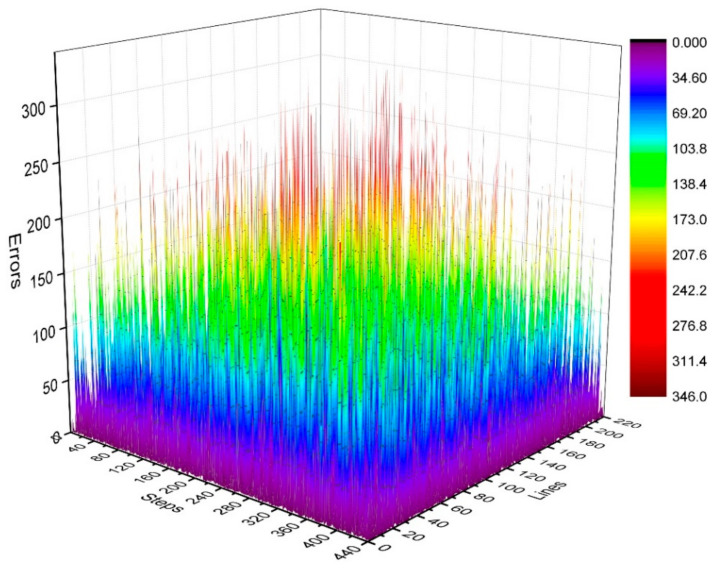
Three-dimensional diagram of phase difference (error) distributions of K_τ−6_ and K_τ−12_.

**Figure 8:**
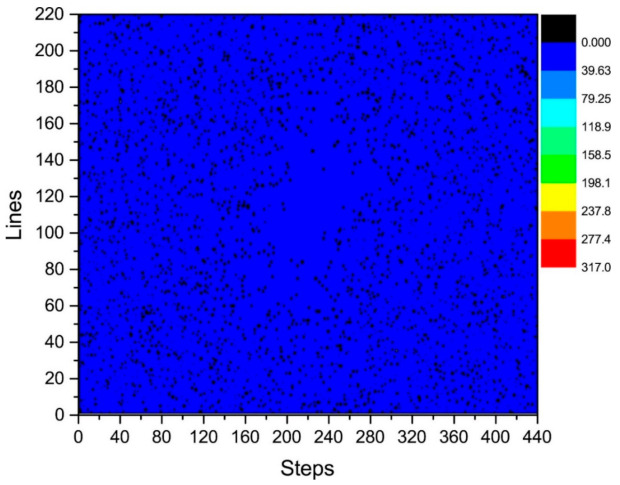
Statistical graph of phase difference (error) distribution in the plane between K_τ−6_ and K_τ−12_.

In K_τ−6_^3^ of K_τ−6_ and K_τ−12_^3^ of K_τ−12_, we defined the phases of the *N* steps in the middle of each phase-encoding line as $\varepsilon_{\left(\tau -6\right)ij}$ and $\varepsilon_{\left(\tau -12\right)ij}$, where i is the number of phase-encoding lines, i.e., i=73, 74,…, 148, j=1, 2,⋯,N. Order:

(8) $$\gamma_{ij}=\varepsilon_{\tau \left(\tau -6\right)ij}-\varepsilon_{\left(\tau -12\right)ij}$$

where i is the number of phase-encoding lines, i.e., i=73, 74, …, 148, j=1, 2,⋯,N. We then calculated the distribution of $\gamma_{ij}$ in the k-space, as shown in Figure 7 and Figure 8. Figure 7 depicts the phase difference (error) distributions of the corresponding positions of K_τ−6_ and K_τ−12_ in degrees (°). Figure 8 illustrates the plane diagram of the statistical distribution of phase difference (error) between signals at K_τ−6_ and K_τ−12_.

By analyzing the regularities in the distribution of phase difference in Figure 4, Figure 7 and Figure 8, we found that the signal amplitude value in the surrounding area of k-space is low, and the signal is greatly affected by noise; therefore, the phase difference of the signal in the area surrounding the k-space fluctuates strongly. The central area has a higher signal amplitude, and the signal is less affected by noise; therefore, the phase difference of the corresponding position signal in the central area tends to be stable (the phase difference approaches 0). As the signal in the central area is less affected by noise, the phase difference of the corresponding position signal in the central area represents the phase difference of the corresponding position signal in the entire k-space; therefore, the phase differences of all corresponding position signals of K_τ−6_ and K_τ−12_ were considered to be 0.

The phase difference between K_τ_ and K_τ−12_ was obtained by adding the phase differences between K_τ_ and K_τ−6_ and K_τ−6_ and K_τ−12_. As observed in Figure 6 and Figure 8, the phase differences between K_τ_ and K_τ−6_ and K_τ−6_ and K_τ−12_ are zero. Therefore, the phase difference between K_τ_ and K_τ−12_ is also zero.

The spliced k-space data are composed of K_τ_^1^, K_τ−6_^2^, and K_τ−12_^3^, and these three areas of data are obtained by scanning each GRE sequence under three different RG parameters. Although, in theory, only the receiver hardware has an RG difference during these three scans, the entire physical state of the MRI may not be completely consistent. Therefore, it is necessary to consider whether there is a phase difference between the three areas of the data during the data splicing process.

The controllable RG circuit of the receiver hardware is composed of a fixed amplifier cascaded with a programmable attenuator; hence, by controlling the programmable attenuator, different RGs can be obtained. In addition, when the receiver hardware is set with different RGs, the signal phase may fluctuate randomly at different levels. Therefore, it is necessary to analyze the signal phase difference under different RGs. The phase difference of the corresponding data of K_τ_ and K_τ−6_ is statistically analyzed in Figure 5 and Figure 6. That of the corresponding data of K_τ−6_ and K_τ−12_ is statistically analyzed in Figure 7 and Figure 8. By calculating the sum of the above two phase differences, the phase difference of K_τ_ and K_τ−12_ is obtained.

### 3.5. Phase Compensation

After gain compensation, the data phase of K^1^ remains unchanged. As per the phase correction results, the phase compensation values of both K^2^* and K^3^* are defaulted to zero, i.e., the phases of the K^2^* and K^3^* data points remain unchanged.

### 3.6. Experimental Design

A commercial 1.5T MR scanner (Alltech Medical Systems LLC, Chengdu, China) was used to verify the effectiveness of the research method proposed in this paper. In the experiment, an 8-channel head coil was used as the signal induction coil. As each channel was distributed in a different spatial position along the head coil, the maximum sensitivity of each channel was determined according to the spatial distance between the coil channel position and the sample, i.e., the surface of the scanned sample adjacent to the coil channel could produce a higher SNR. As the distance between the scanning sample position and the coil channel increased, the SNR of the signal induced by the coil decreased sharply, resulting in the coil channel being insensitive to the received signals. Each channel of the 8-channel head coil has a different degree of sensitivity in different spatial regions of the scanned sample (homogeneous phantom). This experiment sequentially analyzed the data collected by each channel of the 8-channel head coil and verified the proposed method by contrasting the SNR of the image reconstructed from the k-space data on splicing and the SNR of the reconstructed image from the k-space data obtained at the lowest RG. The area with the highest gray value of the scanned sample was selected as the signal area of the region of interest (ROI), and the four corner background areas of the image area were used as the black area. We used the following SNR calculation formula: SNR = SI/SD, where SI is the average signal intensity of ROI, the St Dev of the four black areas was calculated, respectively, then the total St Dev of the four black areas was calculated, which was averaged to obtain SD.

## 4. Results

The improvement of the SNR of k-space signals was explored in this study via optimization of the hardware parameters of MRI. At present, this technology is still in the theoretical verification stage, and the equipment is also in the preliminary testing phase. According to the guidelines for medical equipment research, the device does not currently satisfy the conditions to carry out experiments on humans, because it has not yet been assessed for safety; moreover, animal experiments also require further design. To verify the feasibility of the technology, we therefore designed a uniform water model experiment for initial validation. The actual sampling results of the experiment are shown in Figure 6, and the performance comparison is shown in Table 1.

**Figure 9:**
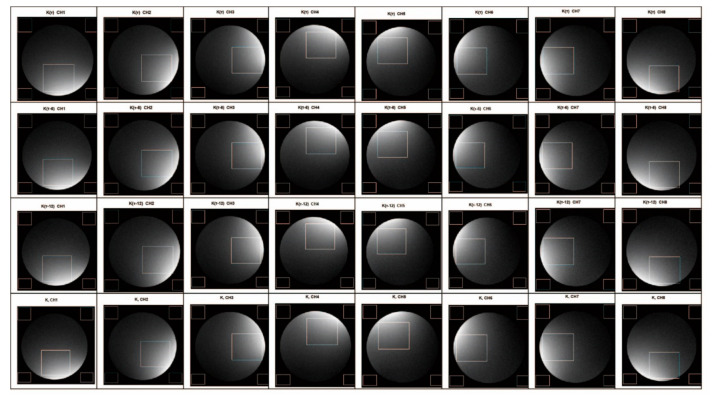
Eight-channel coil subchannel imaging comparison.

To verify the performance of the proposed method, we produced the SNR maps of 32 of the reconstructed images from the eight channels. We took the first slice from each channel and derived a total of 24 images. In Figure 9, 24 images were the reconstructed images from each channel under different RGs (τ dB, τ − 6 dB and τ − 12 dB), and eight were the images of each channel spliced according to the proposed method. In the experiment, we used the following SNR calculation formula that is commonly used in the field of magnetic resonance: SNR = SI/SD, where SI is the average signal intensity of the phantom area of interest, and SD is the average standard deviation from four corner black areas outside the phantom area. Although the black areas may contain ghost signals that increase the standard deviation, thereby over-estimating the noise, such an error would be the same for all of the images, because this experiment is concerned with a comparison of the measured ratio of signal to noise between test cases rather than the intensity of black noise itself. Therefore, the impact of artifacts on black noise does not affect the relative difference of SNR between multiple groups of comparison trials. Table 1 illustrates the improvement of the SNR between the image reconstructed by splicing the k-space data for each channel and the image reconstructed by the k-space data under the lowest RG.

To negate the effect of the difference between the spatial positions of the channel sensitivity, we did not fix the display brightness distribution area on the image. In the analysis, we used the brightest area of the reconstructed image from each channel as the signal area to calculate the changes in the SNR. By comparing the calculation results (Table 1), we found that the SNR of the image reconstructed from the spliced k-space data is significantly higher than that of the image reconstructed from that obtained under the lowest RG (5–13%), demonstrating the effectiveness of the proposed method.

In the actual imaging process of MR equipment, it is necessary to set the K_τ−12_ space to the highest RG value possible for the hardware to obtain the best SNR. This will cause the K_τ−6_ and K_τ_ signals to overflow, leading to the non-existence of K_τ−6_ and K_τ_ signals in the actual sampling process. To obtain the effect of the spliced k-space image, two ideal conditions are assumed here, namely that the signals of K_τ−6_ and K_τ_ do not overflow. K_τ−12_ is a condition that appears in the actual sampling process. The three spaces of K_τ−12_, K_τ−6_, and K_τ_ are spliced to obtain K. Therefore, in the comparative analysis, it is only necessary to compare the data in K with those in K_τ−12_ that can be obtained by the actual equipment.

## 5. Discussion

Table 1 indicates that the SNR difference of the eighth channel in the head coil is relatively small under the RGs of τ − 6 and τ − 12. Therefore, the enhancement of the image SNR corresponding to spliced k-space data of the eighth channel is less evident than that of other channels. This would occur if the actual gain difference between the RGs of τ − 6 and τ − 12 of the eighth channel is smaller than that of the other channels.

References [11,12] discuss the method of improving SNR by adjusting the receiving gain and present an analysis of the SNR improvement effect according to the FID signal of NMR. The method proposed in this paper is mainly intended to improve the image SNR corresponding to k-space data. In reference [13], the effectiveness of the method proposed in this paper was verified by scanning biological structures.

Elliot et al. [19] used water models with structures and teeth to compare the improvement in image detail resolution. Oh et al. [20] used ADC with different resolutions to carry out comparison test. Due to the limitation of current experimental conditions in this paper, we plan to compare our experimental conclusions with those of Elliot’s and Oh’s after we improve our experimental conditions in the next step. Although the quantitative standards of experimental results of previous similar studies are not uniform, they all indicate that the imaging quality was improved. The method proposed in this paper also improves the SNR of MRI images, thus indicating that the proposed gain correction and compensation method is effective. When the RGs are set to other combinations, the k-space data splicing and gain normalization methods presented in this paper are also applicable. In fact, k-space segmentation can also involve more than or less than three areas, but the segmentation into three areas mainly considers the method proposed in this paper through fewer correction and compensation steps.

ADC is a part of the MRI hardware, and ADC quantization noise has a certain impact on image SNR. The main purpose of this paper is to make full use of the ADC resolution through the variable gain in order to improve the SNR of the digital signal after the ADC quantization. When a homogeneous phantom is used for scanning and imaging, the distribution of image black noise is more uniform, and the SNR value can be more accurately calculated using image data. With the same scanning time, a higher SNR results in a higher spatial resolution; if the spatial resolution is the same, the result is a higher temporal resolution. In future work, we will continue to improve the research equipment and use the standard MR resolution phantom for spatial and temporal resolution tests of images.

## 6. Conclusions

To improve the SNR of the received signal, three RGs were designed in this study, and a GRE sequence scan on each RG parameter was conducted to obtain three groups of k-space data. Then, these three groups were spliced into one group. In the splicing process, data obtained under different k-space gain parameters are normalized by the amplitude under the maximum RG parameter. Herein, we also present the methods to correct and compensate for the gain and phase in the normalization process. An SNR comparison between the images reconstructed from the spliced k-space data and the k-space data obtained under the lowest RG was further performed to verify the effectiveness of the proposed k-space data splicing and gain normalization methods. The experimental results indicate that the developed methods improve the SNR by 5–13%, indicating that the method proposed in this paper is effective.

## Figures and Tables

**Table 1:** Signal-to-noise ratio (SNR) improvement statistics.

	Channel	Ch. 1	Ch. 2	Ch. 3	Ch. 4	Ch. 5	Ch. 6	Ch. 7	Ch. 8
K-Space									
K_τ_		22.7630	44.3281	21.6566	19.6930	39.9717	19.4287	10.6366	11.6898
K_τ–6_		21.5626	42.5034	20.4974	18.8234	37.7130	18.9975	10.4391	11.2332
K_τ–12_		19.4104	38.4908	18.2706	17.4597	34.2560	17.1687	9.4805	10.5414
K		21.4548	42.3679	20.5500	19.5114	38.1520	19.3044	10.5835	11.1586
SNR boost value (%)		10.5322	10.0727	12.4756	11.7511	11.3732	12.4393	11.6336	5.8551

## Data Availability

Not applicable.
